# Ultrasonication-mediated nitrogen-doped multiwalled carbon nanotubes involving carboxy methylcellulose composite for solid-state supercapacitor applications

**DOI:** 10.1038/s41598-021-89430-x

**Published:** 2021-05-10

**Authors:** Praveen Kumar Basivi, Sivalingam Ramesh, Vijay Kakani, H. M. Yadav, Chinna Bathula, N. Afsar, Arumugam Sivasamy, Heung Soo Kim, Visweswara Rao Pasupuleti, Handol Lee

**Affiliations:** 1 Department of Chemistry, Sri Venkateswara University, Tirupati, Andhra Pradesh 517502 India; 2 Department of Mechanical, Robotics and Energy Engineering, Dongguk University –Seoul, Pil-dong, Jung-gu, 04620 Seoul, Republic of Korea; 3 Department of Integrated System and Engineering, School of Global Convergence Studies, Inha University, 100 Inha-ro, Nam-gu, Incheon, 22212 Republic of Korea; 4 Department of Energy and Materials Engineering, Dongguk University –Seoul, Pil-dong, Jung-gu, Seoul, 04620 Republic of Korea; 5 Division of Electronics and Electrical Engineering, Dongguk University-Seoul, Pildong-ro 1 gil, Jung-gu, Seoul, 04620 Republic of Korea; 6 PG & Research Department of Chemistry, L. N. Government College, Ponneri, Tamil Nadu 601204 India; 7 Chemical Engineering Area, Central Leather Research Institute (CSIR-CLRI), Adyar, Chennai, 600020 India; 8 Department of Biomedical Sciences and Therapeutics, Faculty of Medicine and Health Sciences, University of Malaysia Sabah, Kota Kinabalu, 88400 Sabah, Malaysia; 9 Department of Biochemistry, Faculty of Medicine and Health Sciences, Abdurrab University, Jl Riau Ujung No. 73, Pekanbaru, Riau 28292 Indonesia; 10 Department of Environmental Engineering, Inha University, 100 Inha-ro, Nam-gu, Incheon, 22212 South Korea

**Keywords:** Chemistry, Materials science

## Abstract

In this study, a novel nanohybrid composite containing nitrogen-doped multiwalled carbon nanotubes/carboxymethylcellulose (N-MWCNT/CMC) was synthesized for supercapacitor applications. The synthesized composite materials were subjected to an ultrasonication-mediated solvothermal hydrothermal reaction. The synthesized nanohybrid composite electrode material was characterized using analytical methods to confirm its structure and morphology. The electrochemical properties of the composite electrode were investigated using cyclic voltammetry (CV), galvanic charge–discharge, and electrochemical impedance spectroscopy (EIS) using a 3 M KOH electrolyte. The fabricated composite material exhibited unique electrochemical properties by delivering a maximum specific capacitance of approximately 274 F g^−1^ at a current density of 2 A g^−1^. The composite electrode displayed high cycling stability of 96% after 4000 cycles at 2 A g^−1^, indicating that it is favorable for supercapacitor applications.

## Introduction

Supercapacitors have attracted significant attention because of they have higher power densities, longer cycle lives, and higher energy densities compared to that of conventional capacitors and batteries^1–3^. Supercapacitor mechanisms can be divided into two types: electrochemical double-layer capacitors (EDLCs) and pseudocapacitors. The EDLC behavior of materials with energy accumulated by the electrostatic adsorption of charges on the surface of the electrode; examples of these are carbon materials such as graphite activated carbon and carbon nanotubes (CNTs)^4–6^. The second mechanism is characterized by (ii) Faradaic capacitors employing metal oxides or conducting polymers that involve electrochemical reactions with excellent specific capacitances and cyclic stabilities^7–9^. Nanostructured materials exhibit higher surface areas and densities, which enhance their electrochemical properties via synergistic effects. Therefore, carbon-based composite electrodes are beneficial for supercapacitors, sensors, electrocatalysts, and gas sensor applications^9–11^.

Carbon nanotubes have been predominantly reported worldwide in the last decades owing to their outstanding mechanical properties, thermal stabilities, and electrical conductivities^12^. CNTs with improved properties have been used as potential materials for supercapacitors in the presence of electrolytes^13^. The electrochemical properties of CNTs have been of great interest in recent years because of their fast recharge, long-term cycling performances, and high power densities^14,15^. Kumar et al.^16^ created an Fe_3_O_4_/RGO nanosheet hybrid electrode for supercapacitor applications. This hybrid composite exhibited a capacitance of approximately 455 F g^−1^ at 8 mV s^−1^ and excellent cyclic stability. In addition, cobalt oxide electrodes on nanocrystalline CNT/polypyrrole (PPy) composites have been used in supercapacitor applications^17^.

Kalam et al.^18^ reported a CNT/PVA gel-based electrode material and showed that at a specific capacitance applied voltage and value of 150 mV s^−1^ and 219 F g^−1^, respectively, it had improved electrochemical properties. Karthika et al.^19^ described the construction of a CNT/PVA gel electrolyte on polyester paper, which showed a specific capacitance of approximately 276 F g^−1^ at 5 mV s^−1^ with excellent retention. Moreover, CMC has favorable properties such as water solubility, biocompatibility, non-toxicity, and chemical stability, which makes it an excellent electrode material for supercapacitor applications^19–21^. Shi et al.^22^ reported a conductive polyaniline (PANI) hydrogel in the presence of bacterial cellulose materials for antimicrobial applications. The microcrystalline cellulose-PPy hydrogel composite via a dendritic network was structured to fabricate the electrode for supercapacitor and sensor applications. Li et al.^23^ reported a CMC/PANI composite hydrogel, which acquired high conductivity and excellent mechanical properties^23^. In this study, the porous materials of nitrogen-doped multiwalled carbon nanotubes/carboxymethylcellulose (N-MWCNT/CMC) composites were fabricated using a hydrothermal process for symmetric and asymmetric supercapacitor applications. The composite was confirmed using structural, morphological, and electrochemical applications in the presence of an electrolyte.

## Experimental section

### Materials

Analytical grade Na-CMC with a number-average molecular weight of 90,000, MWCNTs, urea, nitric acid, potassium permanganate, ammonium hydroxide, ethanol, potassium hydroxide, and double-distilled (DD) water were purchased from Sigma-Aldrich Pvt. Ltd. (Korea). The composite materials were synthesized for supercapacitor applications.

### Methods

#### Synthesis of N-MWCNT composite

The MWCNTs were synthesized based on previous studies^24^. The nitrogen doping process of the MWCNTs is shown in detail. The estimated amounts of 2 and 2.4 g of MWCNTs and urea, respectively, were dispersed in 200 mL of DD water, and the mixture was continuously sonicated for 4 h to complete the dispersion. The resultant solution was heated at 80 °C for 6 h and then purified using ethanol/water (1:1). The reaction mixture was then purified at 90 °C in a vacuum oven for 24 h to analyze its structural, morphological, and electrochemical properties.

#### Synthesis of the N-MWCNT/CMC composite

**Figure 1:**
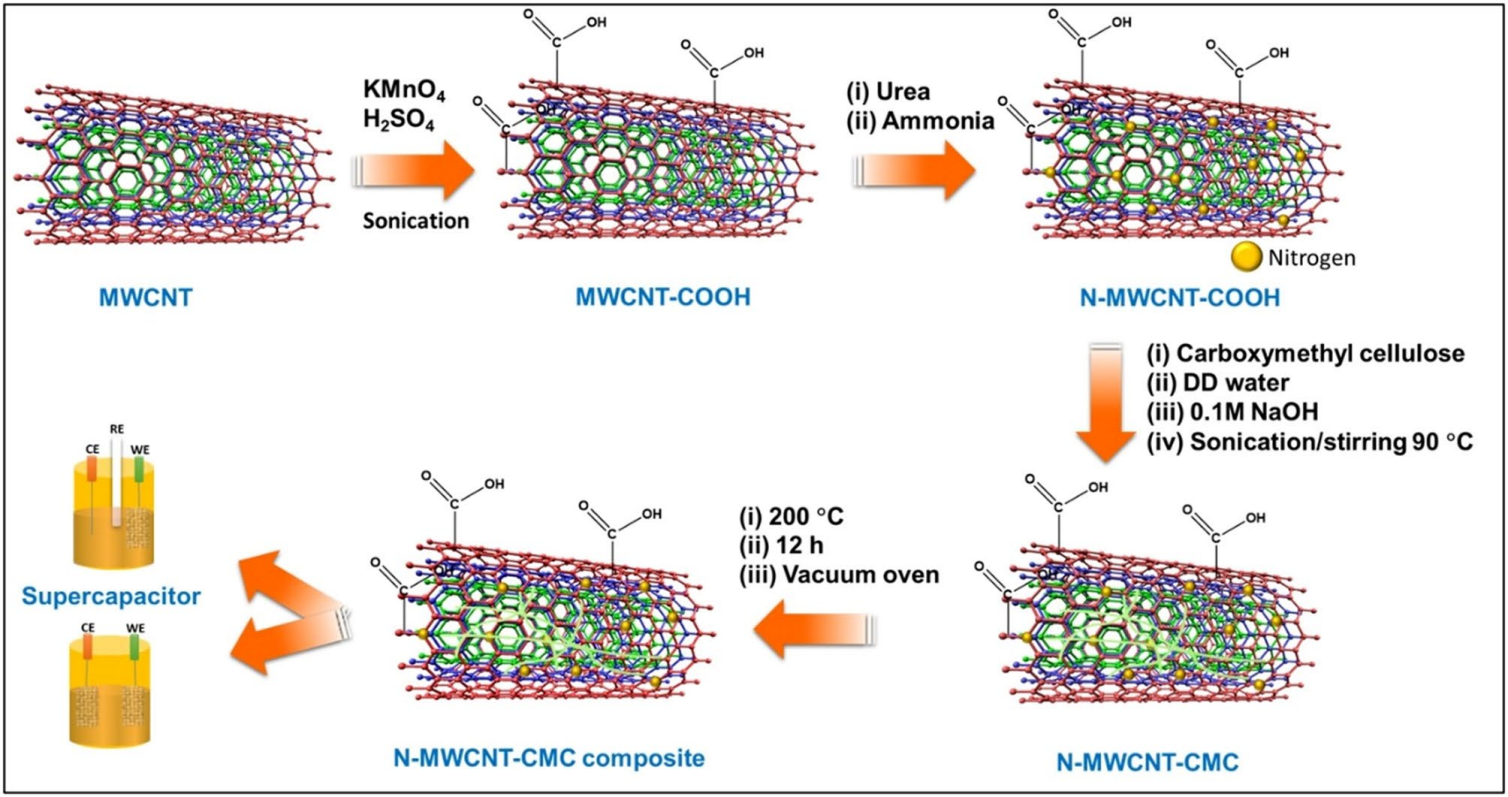
Schematic illustration of the N-MWCN/CMC composite.

A mixture of N-MWCNT (0.5 g) and CMC (2 g) in 200 mL of DD water was vigorously stirred at 95 °C for 4 h. Subsequently, 20 mL of ammonia was added to the reaction solution, and the solution was stirred at the same temperature for another 12 h. The reaction mixture was purified using ethanol at 200 °C for 12 h using a vacuum oven. The resultant product was transferred to an alumina crucible and heated at 400 °C for another 8 h to complete the formation of the hybrid composite, which was ready to be characterized. The synthesis process of the N-MWCNT/CMC composite is described in Fig. 1.

#### Materials characterization

Fourier-transform infrared spectroscopy (FTIR, Model number, PerkinElmer) was used to analyze the functionality of the N-MWCNT/CMC composite. Raman spectral examination of the composite was conducted using an RM200 confocal Raman microscope (alpha300R). The nanostructures of the composite were ascertained using the powder X-ray diffraction (XRD) patterns (Rotaflex RU-200B, RIGAKU) and a source of Cu Kα radiation. X-ray photoelectron spectroscopy (XPS) analysis of the elemental composition was conducted using an ESCALAB 250Xi instrument in the presence of Al Kα radiation. The morphologies of the composites were characterized using field emission scanning electron microscopy (FE-SEM, Hitachi S-4800, Japan) and high-resolution transmission electron microscopy (HR-TEM, JEM-2010F, Japan).

#### Electrochemical measurements

The CV results of the composite were characterized using a three-electrode configuration containing Pt, Ag/AgCl, and working electrodes (N-MWCNT/CMC). Electrochemical analysis was performed using CV, GCD, and EIS, and was conducted on an Auto lab PGSTAT302N in Metrohm, Netherlands. The (75:20:10) electrode material was fabricated using polytetrafluorethylene and forced into a current collector of nickel wire (1 cm × 1 cm). The electrochemical properties of the N-MWCNT/CMC composite electrode were characterized using cyclic voltammetry (CV), galvanostatic charge/discharge (GCD), and electrochemical impedance spectroscopy (EIS) analysis via a 3 M KOH electrolyte. The electrochemical properties of the N-MWCNT/CMC composite electrode were characterized using CV, GCD, and EIS analyses. The CV was conducted in a potential range between 0.0 and 0.6 V versus Ag/AgCl at different scan rates in the presence of an electrolyte. The constant current charge-discharge test was conducted at different current densities of 2 to 6 A g^−1^ in the potential range of 0.1 to 0.6 V. The EIS results were obtained to confirm the capacitive performance at an open circuit potential of 0.01–105 Hz.

### Results

**Figure 2:**
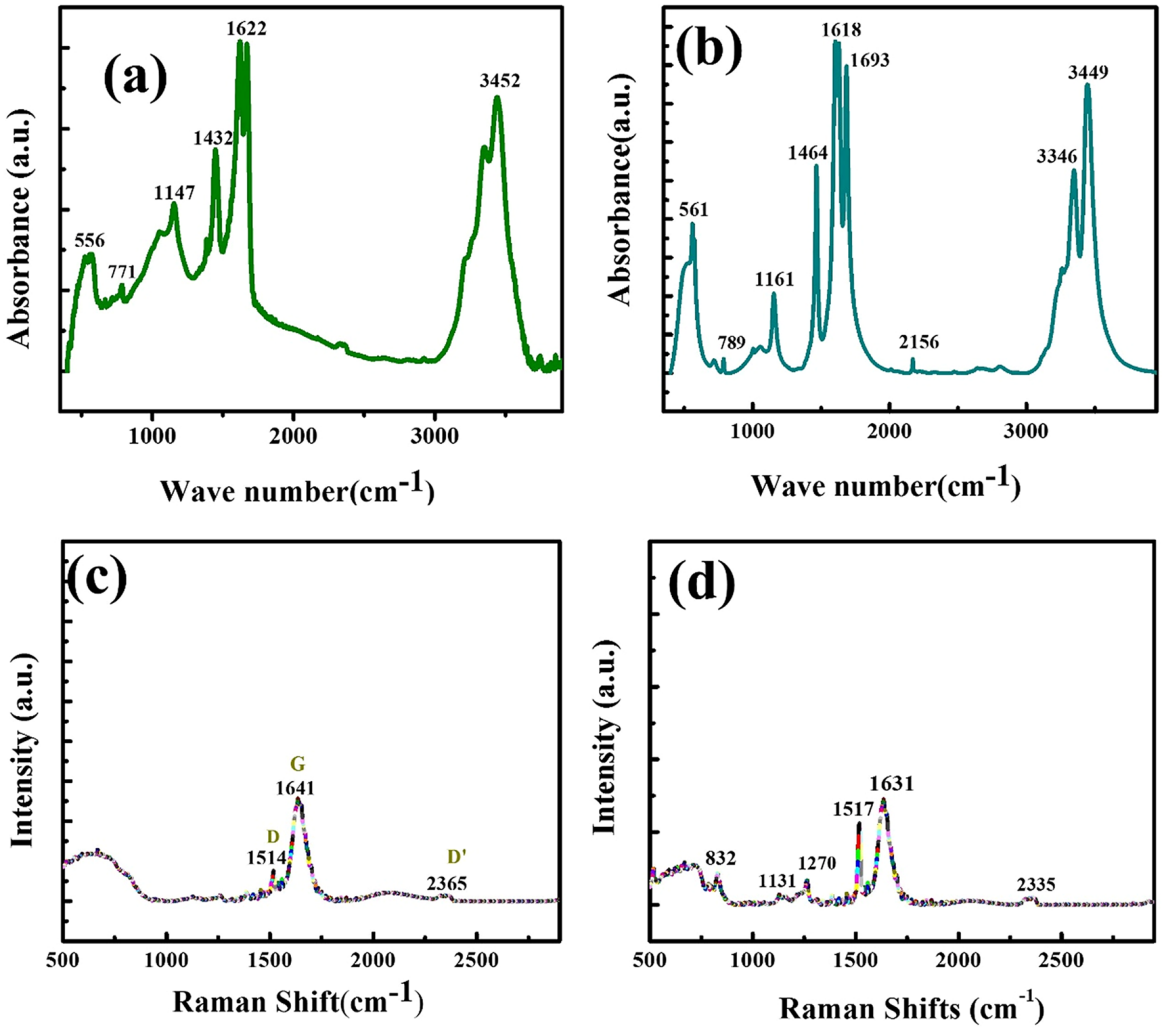
(**a**,**b**) FTIR and (**c**,**d**) Raman results of the N-MWCNT and N-MWCNT/CMC composites.

Figure 2a,b show the FTIR results of the N-MWCNT and N-MWCNT/CMC composites, respectively. The peaks at 3452, 1622, 1452, 1147, and 771–556 cm^−1^ represent the carboxylic functionalized groups on the nitrogen-doped MWCNT composite. Fig. 2b shows the peaks at 3449, 3346, 2156, 1693, 1618, 1464, 1161, and 789–561 cm^−1^, which represent the N-MWCNT/CMC composite. Raman spectral analysis was employed to investigate the N-MWCNT/CMC composite and its defect arrangement. Figure 2c shows the Raman spectra of the composite via a sonication-mediated hydrothermal process. The peak at 1579 cm^−1^ represents the G peaks of *sp*^2^ carbon atoms, and the D band at 1342 cm^−1^, which was assigned to the breathing modes of the A1g symmetry^25^, represents the local defects and disorder in the N-MWCNT materials. The peaks at 1342 (D band), 1579 (G bands), and (2684−2931) cm^−1^ (2D and 2D’) results are shown in Fig. 2d. The intensity proportion of the D to G band is approximately 1.03, which indicates that most of the oxygen functional groups were intercalated into the N-MWCNT nanotubes via the hydrothermal reduction process^26^.

**Figure 3:**
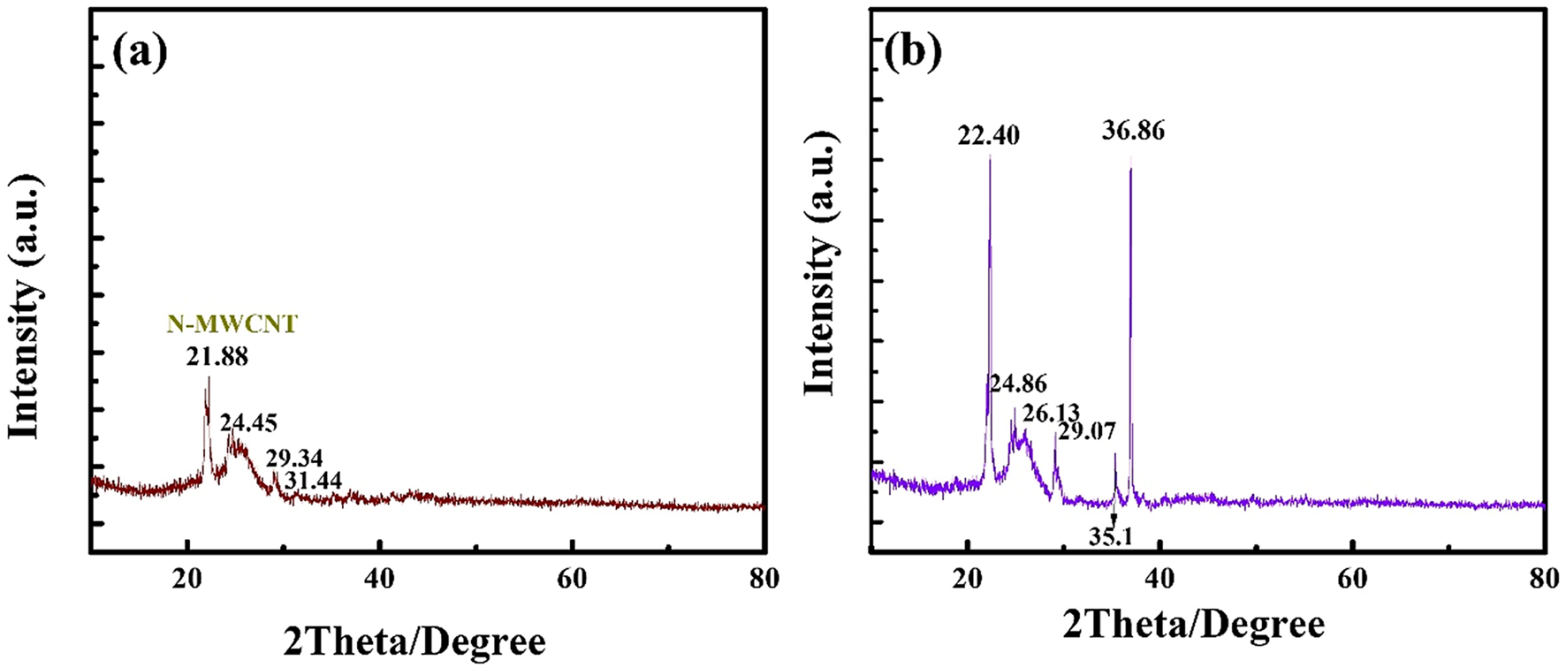
XRD results of the (**a**) N-MWCNT and (**b**) N-MWCNT/CMC composites.

The XRD analysis of the MWCNT and N-MWCNT nanostructured composites and structural and morphological information have been reported in the literature^27^. The XRD results of the N-MWCNT and N-MWCNT/CMC composites are shown in Fig. 3a,b, respectively. The peaks at 21.88°, 24.45°, 29.34°, and 31.44° in Fig. 3a correspond to the (002), (100), (004), and (110) planes of the N-MWCNT composite, respectively. Figure 3b shows that the peaks at 22.40°, 24.86°, 26.13°, 29.07°, 35.10°, and 36.86° represent the planes of the N-MWCNT/CMC composite, which is similar to the N-MWCNT case, i.e., the (002), (100), (004), and (110) planes.

**Figure 4:**
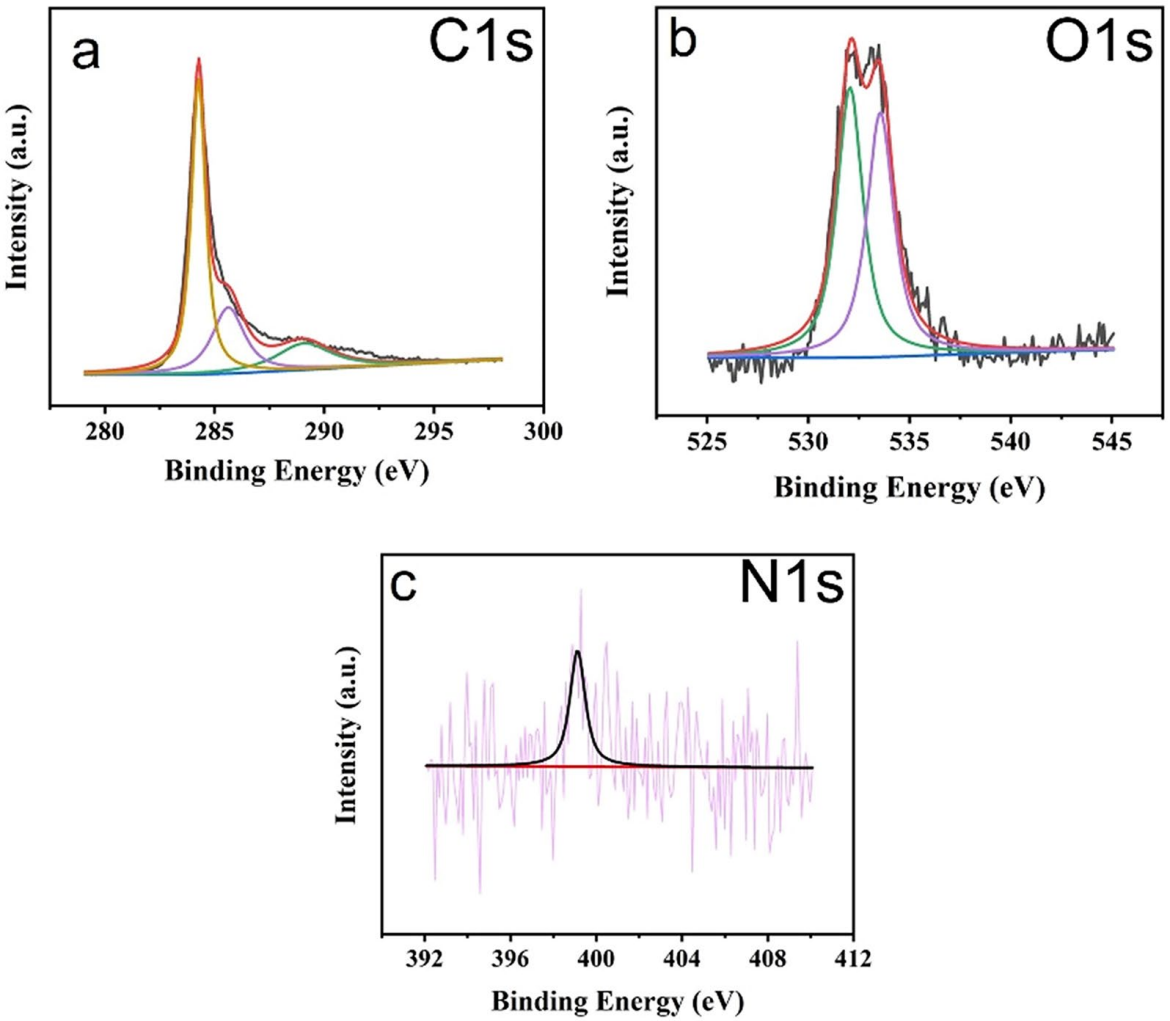
XPS results of the N-doped MWCNT composite.

**Figure 5:**
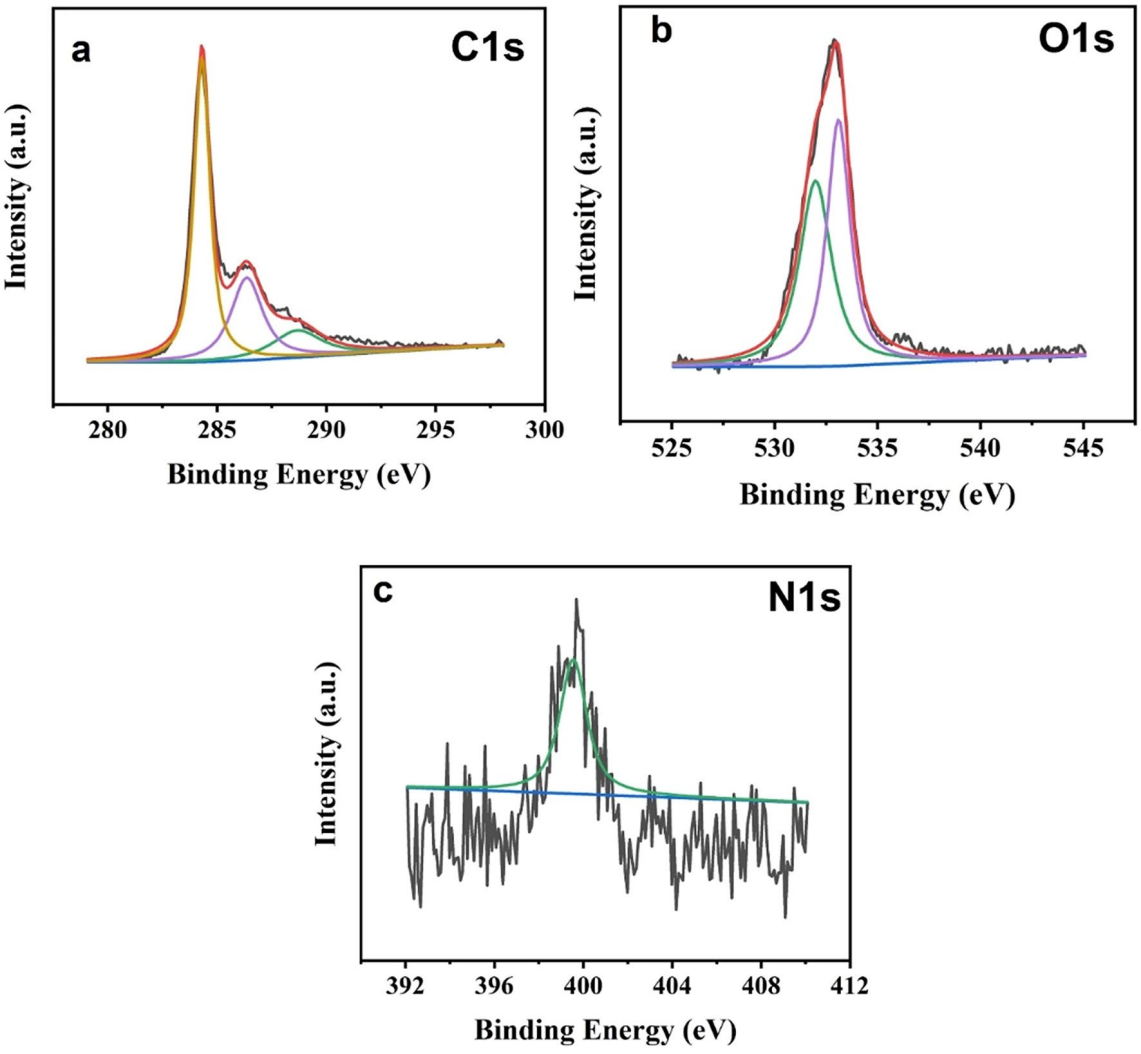
XPS results of the N-doped MWCNT/CMC composite.

XPS was used to study the elemental composition of the N-MWCNTs, and the composite results are displayed in Figs. 4 and 5. It displays the elemental structure and chemical states of the N-MWCNT and composite peaks at C1*s* (284), O1*s* (531), and 395–408 (N1*s*). The typical peaks of C, O, and N were confirmed in the N-MWCNT composite, and the XPS fitting figures are shown in Figs. 4 and 5, which confirm the N-MWCNT and N-MWCNT/CMC composite nanostructured materials, respectively. The doping concentration is very low due to the N elements are shown in noise signal.

**Figure 6:**
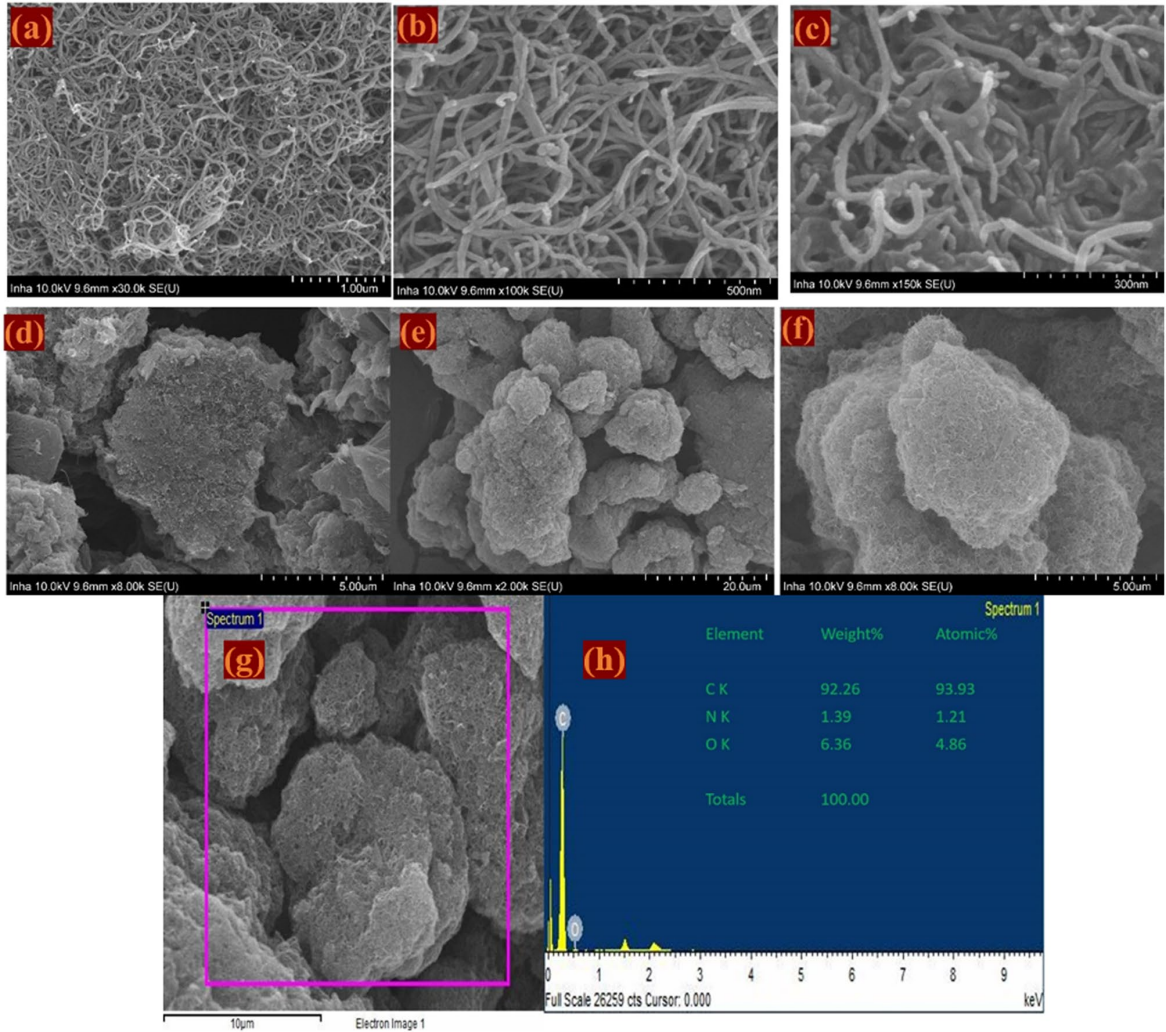
SEM images of (**a**–**c**) N-MWCNT and (**d**–**f**) N-MWCNT/CMC composite in different magnifications, and (**g**,**h**) EDS analysis of the N-MWCNT/CNC composite.

The morphological analysis of the N-MWCNT and N-MWCNT/CMC composites was conducted using FE-SEM, and FE-SEM EDS results are displayed in Fig. 6. Based on the SEM results, small globular particles, such as cauliflower, accumulated and were allocated on the N-MWCNT surface on a nanometer scale. The SEM-EDX results suggest that the composition of elements such as carbon (93.93%), oxygen (4.86%) and nitrogen (1.21%) is successfully doped via hydrothermal reaction. These well-organized composites are assembled with a higher surface area and smaller diffusion, which can improve the electrochemical properties of the composites^28–30^.

**Figure 7:**
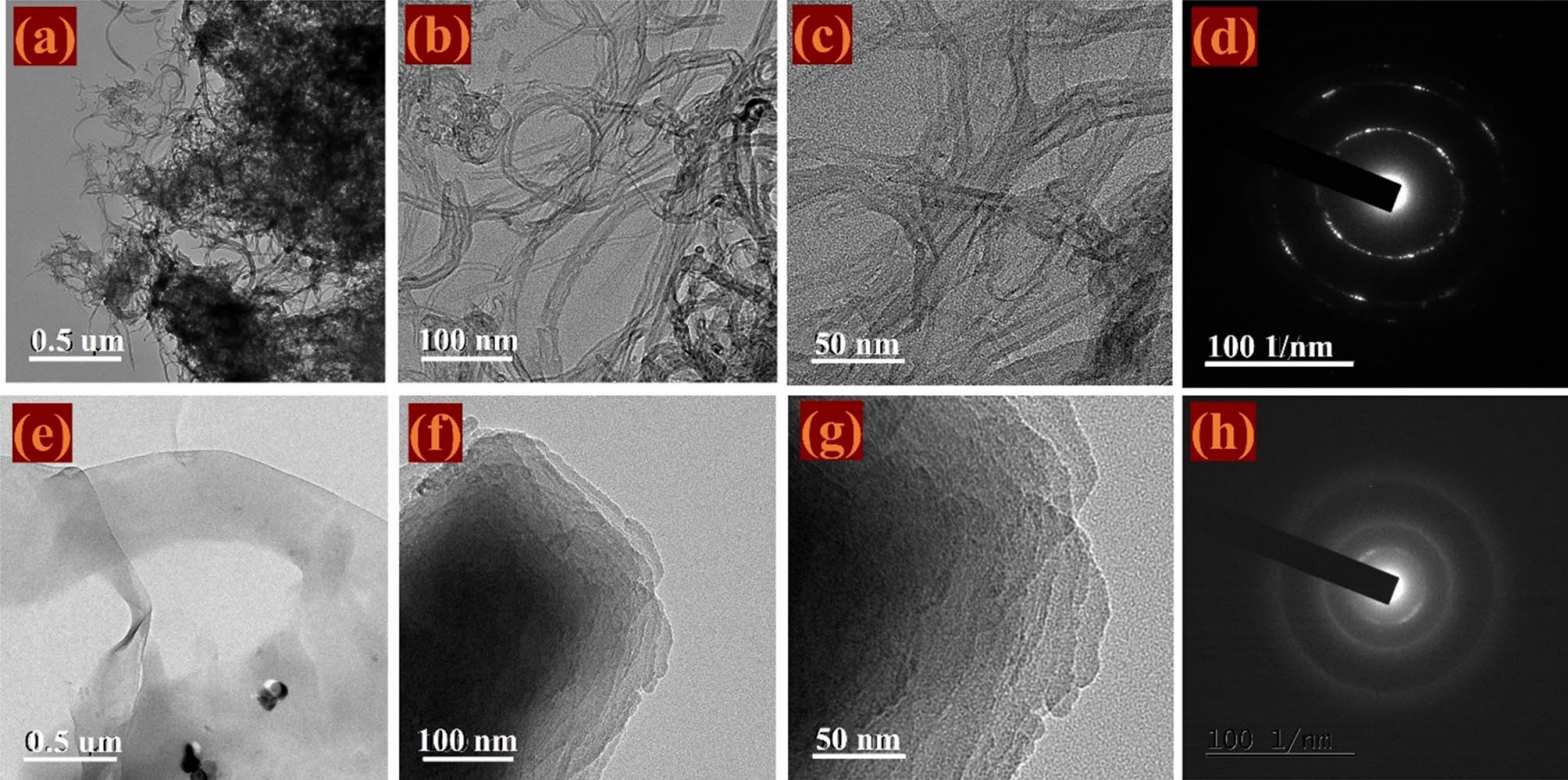
TEM images in different magnifications and SAED analysis of composites, respectively: (**a**–**c**) and (**d**) for N-MWCNT; (**e**,**f**) and (**h**) for N-MWCNT/CNC.

The morphologies of the N-MWCNT and N-MWCNT/CMC composites, which were examined using HR-TEM are shown in Fig. 7. Based on the TEM images, the composite contained bundles of nanotubes and exhibited a crystalline behavior of the CMC in the range of approximately 20–100 nm. Moreover, nitrogen was predominant on the surface of the nanotubes. The N-MWCNT/CMC composite is a well-defined nanostructure with carbon nanotubes at the nanometer scale and sheet-like morphology, as shown in Fig. 7d. Therefore, the N-MWCNT/CMC composite with a conductive core improved the remarkable properties shown in the electrochemical analysis. The selected area electron diffraction (SAED) pattern of the composite is shown in Fig. 7, which shows the nanocrystalline behavior of the N-MWCNT and composite samples. The results indicated that the precise rings proved the nano polycrystalline morphologies of the N-MWCNT and CMC composite.

#### N-MWCNT/CMC composite for electrochemical CV analysis

**Figure 8:**
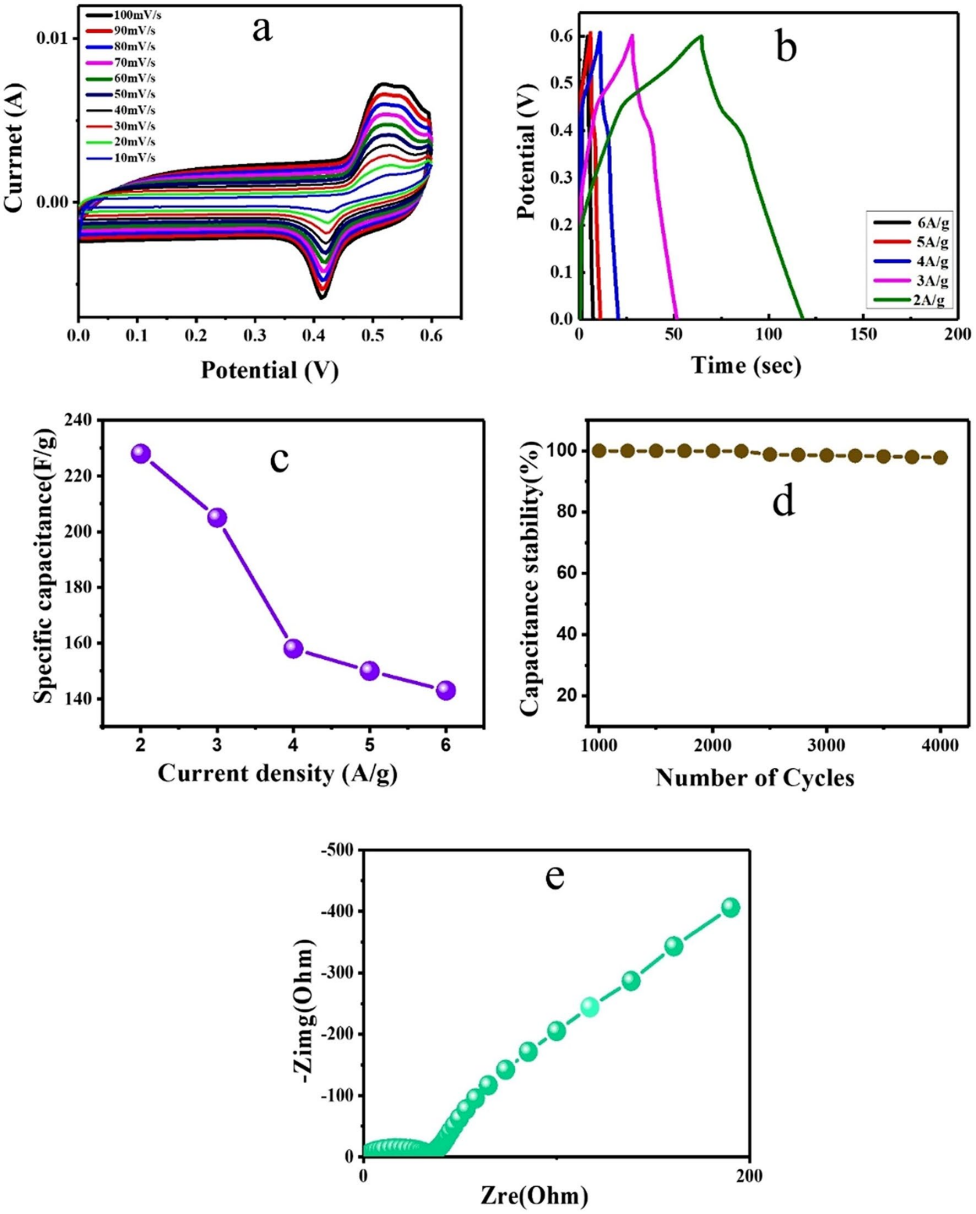
CV analysis of the N-MWCNT/CMC composite for the three-electrode configurations via 3 M KOH solution.

**Figure 9:**
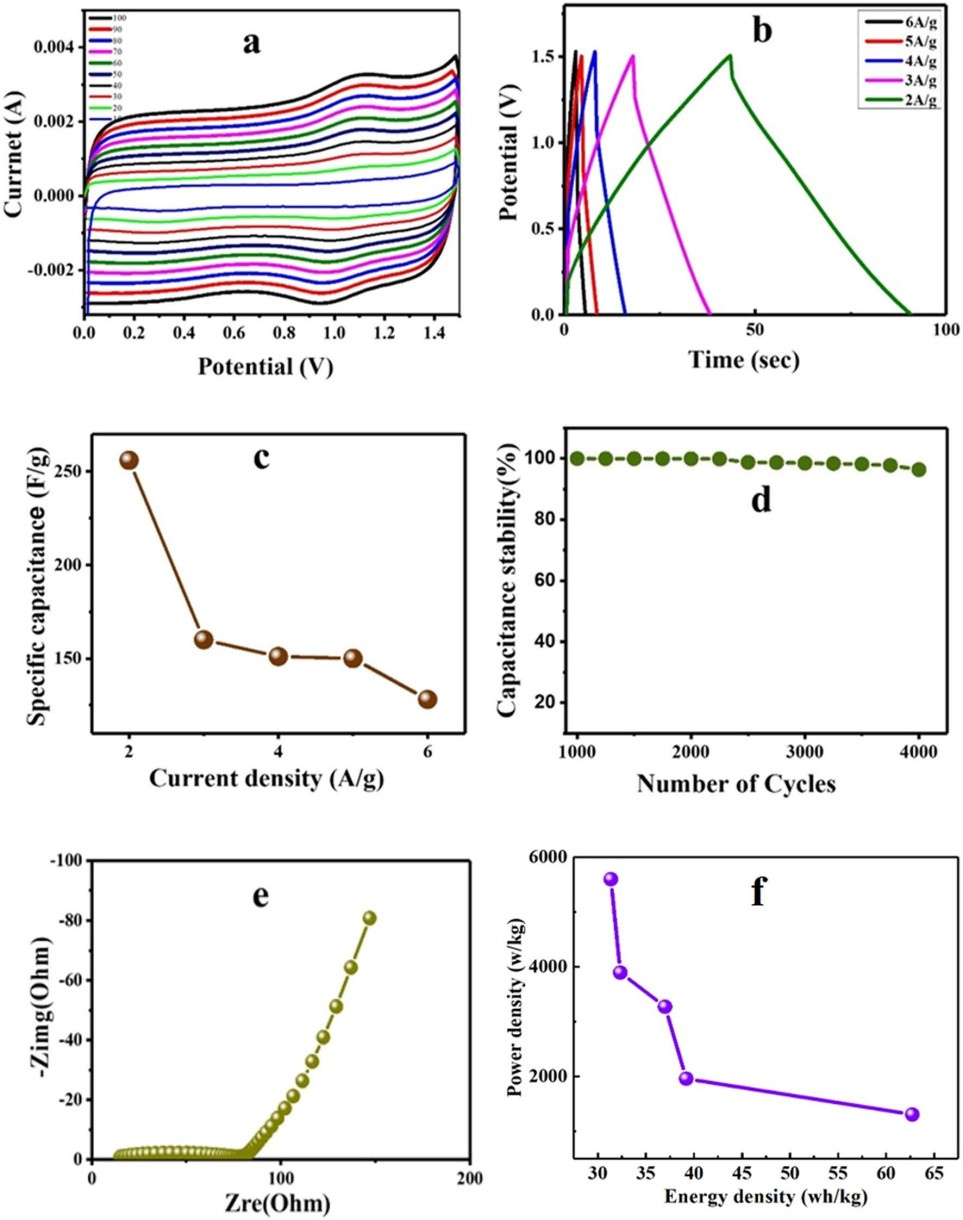
CV analysis of the N-MWCNT/CMC composite for the two electrode configurations via 3 M KOH solution.

The CV results of MWCNT and N-MWCNT materials are widely used to fabricate electrode materials for supercapacitor applications^15^. The N-MWCNT/CMC electrode was used to produce two and three-electrode configurations for electrochemical applications. In this study, the CV, GCD, and EIS results were investigated using a three-electrode setup in a 3 M KOH aqueous electrolyte. In this study, the Pt wire, Ag/AgCl, and N-MWCNT/CMC composites were used as the counter, reference, and working electrodes, respectively. The results of the CV analysis of the N-MWCNT/CMC composite via three and two-electrode configurations are shown in Figs. 8 and 9, respectively. In the CV experiment, various scan rates of 10, 30, 50, 70, 90, and 100 mV s^−1^ in the potential range of 0 to 0.6 V in 3 M KOH aqueous solution were used. Figures 8a and 9a show the rectangular peaks, indicating an electrical double layer capacitance (N-MWCNT) and pseudo capacitance from CMC. The results represented the CV analysis conducted at various scan rates in 3 M KOH electrolyte over the potential range of 0–0.6 V versus Ag/AgCl for supercapacitor behaviors. The CV curves of the composite electrodes were shown in rectangles with redox peaks in their electrochemical behaviors. The active mass and resistance of the electrode increased because the geometrical area of the electrode exhibited a similar behavior. The N-MWCNT/CMC composite electrode showed a higher current response than that of pristine N-MWCNT, indicating an excellent electrochemical performance. These results revealed that the total capacitance of the electrode increased owing to the incorporation of the CMC matrix on the CNTs. The CV results of the composite electrode (Fig. 9a) at various scan rates indicated an excellent electrochemical supercapacitor behavior.

Figure 8b shows the GCD curves of the N-MWCNT/CMC composite electrodes in the presence of 3 M KOH solution at current densities of 2, 3, 4, 5, and 6 A g^−1^. The results indicated that an approximately similar potential-time behaviors occurred, which indicated that the GCD process of the composite sample was a reversible reaction in terms of their electrochemical properties^31^. The specific capacitance of the composite materials was calculated from the following equation^32^: C (F g^−1^) represents the specific capacitance, I (A) is the galvanostatic current used for GCD, t (s) is the time denoted as the discharge cycle, V (V) is the potential difference of the discharge, and m (g) is the mass of the electrode material. Therefore, the composite materials were subjected to superior capacitance (~228 F g^−1^) than that of the pristine N-NMWCNT materials. The significant improvement in the specific capacitance of the composite changed the size of the nanoparticles, redox activity, and electronic conductivity of the CNT and CMC. There are numerous works on conducting polymers involving CMC composites and carbon materials that were used in supercapacitor applications^32^. It was reported that the capacitance of PANI/CMC can reach up to 451.25 F g^−1^ by employing 20wt% CMC composites in conjunction with carbon materials. Also, they reported that at a current density of 1 A g^−1^ exceeding 1000 cycles, the capacitance reaches up to 300 F g^−1^. Similarly, other work specified that the capacitance of PPy/CMC nanospheres can reach up to 184 F g^−1^ by employing at current density of 0.25 Ag^−1^. Additionally, the 80% retentive cyclic stability has attained for a hybrid electrode with CMC template after 200 cycles which is efficient for the supercapacitor applications^33^. Babu IM et al.^34^ described on well-ordered mesoporous Co_3_O_4_/CMC nanoflake assembly likewise provides limited diffusion route for ions and hasten operative charge carriage that reflects in elevated capacitance of 298 °C g^−1^ at 1 A g^−1^, exceptional stability of 90% maintenance after 5000 cycles and little charge passage resistance of 0.5 Ω in three electrode schemes. Moreover, they reported the design of asymmetric supercapacitor operating at voltages 0–1.2 V which exhibits appropriate electrochemical performance at 2 A g^−1^ with an energy density of 18 W h kg^−1^. These results support the prospective competencies of incorporated Co_3_O_4_ & CMC which could be employed as an efficient electrode in the forthcoming supercapacitor applications.

The composite electrode showed a maximum capacitance of approximately 228 F g^−1^ at a current density of 2 A g^−1^, indicating an exceptional rate capability in the presence of a 3 M KOH electrolyte. Figure 8c shows the plot of the specific capacitance (Vs) at various current densities. The capacitance of the composite decreased from approximately 228, 205, 158, 150, and 143 F g^−1^, and the current density varied from 2 to 6 A g^−1^. Figure 9d shows the cyclic retention of the composite at 2 A g^−1^ for 4000 cycles and 4% capacitance loss. Moreover, it shows the excellent stability of the composite in the presence of 3 M KOH. Figure 8e shows the Nyquist plots of the composite materials. The result depends on the perpendicular line and semicircle in the low and high-frequency regions (0.1–100 Hz), respectively. The subsequent (Fig. 9e) simple equivalent circuit requires solution resistance (Rs), charge transfer (Rct), double-layer capacitance (Cdl), and pseudo capacitance (Cp). Therefore, the perpendicular line in the low-frequency region denotes the electrochemical behavior and fast diffusion of electrolyte ions in the composite material, and the semicircle diameter implies Rct. The Rct of the electrodes of the 1st and 1000th cycles are approximately 0.9 and 35, respectively, affecting the stable electrochemical properties. The phase angle for the impedance plot of the composite electrodes was observed to be higher than 45° at low frequencies, suggesting electrochemical capacitive properties. Therefore, the results showed that the composite electrode was favorable for supercapacitor properties^35–51^.

### Discussion

The N-MWCNT/CMC composite was studied using a two-electrode system containing a PVA/H_2_SO_4_ gel electrolyte for supercapacitor applications, as shown in Fig. 9. The CV curves of the composite electrode were obtained at various scan rates of 10, 20, 30, 40, 50, 60, 70, 80, 90, and 100 mV s^−1^. As seen in Fig. 9, the CV curves are approximately rectangular, which indicates the excellent electrochemical properties and fast redox reaction of the composite electrode in the presence of the PVA/H_2_SO_4_ gel electrolyte. Figure 9b shows that the GCD test was conducted for the device at current densities of 2, 3, 4, 5, and 6 A g^−1^ with corresponding specific capacitances of approximately 256, 160, 151, 132, and 128 F g^−1^, respectively. Before the GCD cycles, the EIS test was conducted in a frequency range of 0.01–105 Hz, which demonstrated an internal resistance of 9.68 Ω and increased after 4000 cycles to Rct-65 Ω, as shown in the Nyquist plot in Fig. 9d. The results showed that the equivalent series circuit was used for the electrochemical properties. Furthermore, the GCD cyclic stability test was conducted at a 2 A g^−1^ scan rate of up to 4000 cycles, which confirmed that it retained 96% of the initial capacity. Based on the mechanical strain, the specific capacitance value decreased by a 4% loss in capacitance, and the remaining 96% retained its initial capacitance with a coulombic efficiency of approximately 98% of the overall cyclic stability.

## Conclusion

In conclusion, the present work proved that a sonication-assisted hydrothermal process can synthesize an N-MWCNT/CMC composite. The synthesized composite materials exhibited excellent morphologies and porosities, which improved their specific capacitance values. The electrochemical properties of the composite materials showed the highest specific capacitance values of approximately 274 F g^−1^ at a current density of 2A g^−1^ in the presence of 3 M KOH and PVA/H_2_SO_4_ gel electrolytes. This improvement in specific capacitance values was attributed to the excellent penetration and enhanced utilization of the active surface, which is favorable for supercapacitor applications. The excellent structural, cyclic, and electrochemical properties at a higher current rate with approximately 96% cyclic stability were useful in fabricating a wide range of supercapacitor applications. This further proved that composite materials can be used in real supercapacitor applications using two-electrode performance via a PVA/H_2_SO_4_ gel electrolyte. Therefore, the composite materials can be synthesized at a low cost and the electrodes can be efficiently fabricated for promising supercapacitor applications in the future.
